# Chemical Profiling, Quantitation, and Bioactivities of Ginseng Residue

**DOI:** 10.3390/molecules28237854

**Published:** 2023-11-29

**Authors:** Shengyu Ge, Jinlong Liu, Yang Liu, Jiaqi Song, Hongfeng Wu, Lele Li, Heyun Zhu, Bo Feng

**Affiliations:** 1 School of Pharmacy, Jilin Medical University, Jilin 132013, China; gsy19981105@163.com (S.G.); 15584519698@163.com (J.L.); 19888483849@163.com (Y.L.); 18844408299@163.com (J.S.); 18343509083@163.com (H.W.); fengbo10@126.com (B.F.); 2 School of Pharmacy, Yanbian University, Yanji 133002, China

**Keywords:** ginseng residue, ginsenosides, ginseng polysaccharides, UPLC-Q-TOF/MS, antioxidant activity

## Abstract

Ginseng residue is a by-product stemming from the commercial extraction of ginsenosides. To assess the disparities between ginseng residue and ginseng tablet, we employed the ultra-high-performance liquid chromatography–quadrupole time-of-flight/mass spectrometry (UPLC-Q-TOF/MS) technique for sample analysis. The analyses revealed the presence of 39 compounds in both ginseng residue and ginseng tablets. Subsequently, the contents of total ginsenosides and total ginseng polysaccharides in the ginseng residue and ginseng tablet were determined. The results indicate that while only a small fraction of ginsenosides remained in the ginseng residue, a significant amount of polysaccharides was retained. Furthermore, our evaluation encompassed the antioxidant activities of both ginseng residue and ginseng tablets. Notably, ginseng residue exhibited robust antioxidant effects, thereby showcasing its potential for recycling as a functional food raw material.

## 1. Introduction

Ginseng, the roots and rhizomes of Panax ginseng C.A. Meyer, has been used as a herbal remedy or tonic food to rebalance the human body in China, Korea, and Japan for thousands of years [1,2]. Previous studies have reported that ginseng possesses antioxidant, anticancer, hepatoprotective, antidiabetic, anti-obesity, and anti-inflammatory properties [3,4]. Due to its unique bioactivity and pharmacological effects, ginseng is widely used in the clinical treatment of diseases. Additionally, there are many ginseng functional foods, health supplements, and cosmetics available on the market [5,6,7,8,9]. Ginsenosides and polysaccharides are key compounds believed to contribute to the majority of ginseng’s bioactivities [10,11].

Ginseng residue is a by-product of commercial ginsenoside extraction, and its yield has been increasing due to the popularity of tonic foods and drinks. It is estimated that tens of thousands of tons of ginseng residues are generated annually worldwide [12]. In China, ginsenoside extract is typically obtained from ginseng tablets using an ethanol solution of a specific concentration, followed by drying. Ginseng residue contains certain ginsenosides and polysaccharides that exhibit biological activity [13]. Consequently, ginseng residue has the potential to be utilized as a raw material for tonic food development. However, the lack of systematic research on its material basis and bioactivity has limited the recycling and utilization of ginseng residues.

Various analytical tools are now available for the analysis of traditional Chinese medicine, such as high-field NMR [14], liquid chromatography (LC) [15], and mass spectrometry (MS) [16]. Compared with other analytical tools, LC-electrospray-mass spectrometry (ESI-MS) can achieve much higher sensitivity and selectivity and identify chemical compounds in complex matrixes with only a small amount of the sample. Quadrupole time-of-flight mass spectrometry with extremely high resolution, sensitivity, and mass accuracy is a powerful technique for the detection of ginsenosides, especially in small amounts [17]. Thus, the ultra-high-performance liquid chromatography–quadrupole time-of-flight/mass spectrometry (UPLC-Q-TOF/MS) technique was used for the chemical profiling of ginseng residue in this study. The detection principle of the ABTS^+^ free radical scavenging activity assay method is that under the action of oxidants, ABTS is oxidized to green ABTS^+^. The generation of ABTS^·+^ is inhibited in the presence of antioxidants. The absorbance of ABTS^·+^ at 734 nm or 405 nm can be measured to determine and calculate the total antioxidant capacity of the sample. The ABTS^+^ free radical scavenging activity assay is commonly used to reflect the in vitro antioxidant activity of drugs and has been widely applied in the research of Chinese herbal medicine [18].

In this study, we aim to evaluate the qualitative and quantitative differences between ginseng residue and ginseng tablets. To assess the antioxidant activity of ginseng residue, ABTS^+^ radical scavenging activities were conducted. The findings of this research may serve as a valuable reference for researchers and companies seeking to recover and utilize ginseng residue effectively.

## 2. Results and Discussion

### 2.1. Analysis of Chemical Composition in Ginseng Residue Using UPLC-Q-TOF/MS

**Figure 1:**
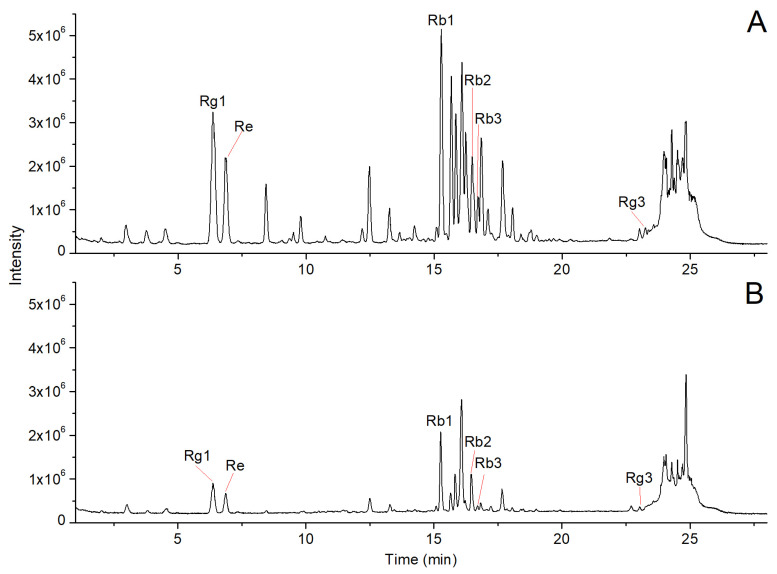
Representative TICs of ginseng tablet (**A**) and ginseng residue (**B**).

In this study, we utilized a UPLC-Q-TOF/MS method to analyze the chemical constituents of both ginseng residue and ginseng tablets. The total ion chromatograms (TIC) of the ginseng tablet and ginseng residue are presented in Figure 1A,B, respectively. It was observed that the compounds in ginseng tablets and ginseng residue were well separated within 30 min in Figure 1, which indicated that the developed UPLC-Q-TOF/MS method is suitable for the analysis of the ginseng tablet and residue samples. As shown in Figure 1A,B, the signal intensities of various chromatographic peaks in the TIC of ginseng tablets were generally higher than the corresponding peaks in the TIC of ginseng residue. Based on the comparison of retention time and exact *m*/*z* with standard substances, six ginsenosides, Rg1, Re, Rb1, Rb2, Rb2, and Rg3, were identified in Figure 1. The signals of these six ginsenosides in the total ion current chromatogram of ginseng residue corresponding peaks showed a significant decrease.

**Figure 2:**
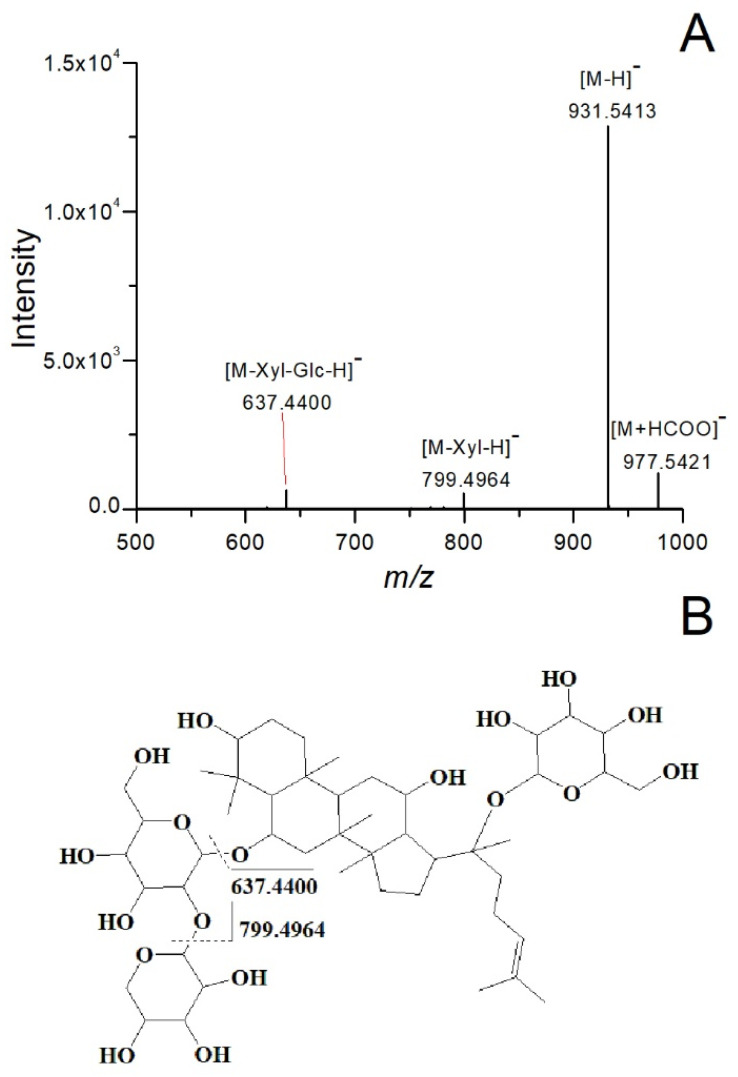
The MS/MS spectrum (**A**) and possible fragmentation pathways of notoginsenoside R1 ion (**B**).

The UPLC-Q-TOF/MS approach allows for the acquisition of accurate *m*/*z* and chemical structure of the compounds through MS and MS/MS techniques. Accurate *m*/*z* of precursor ions, fragment ions, reference mass spectrum data, and the relevant literature reports [2,19,20] were used to further confirm the compounds. Notoginsenoside R1 was used as an example to demonstrate identification procedures. The [M+HCOO]^−^ ion of notoginsenoside R1 at *m*/*z* 977.5421 was selected as a precursor ion. Additionally, the MS/MS spectrum of notoginsenoside R1 is shown in Figure 2A. As shown in Figure 2A, the product ion with the highest signal intensity (*m*/*z* 931.5413) results from the loss of HCOOH, and product ions at *m*/*z* 799.4964 and 637.4400 correspond to the loss of xylose or glucose radicals. The possible fragmentation pathways for the product ions are shown in Figure 2B. Additionally, other ginsenosides were confirmed using the same identification method.

Comprehensive information regarding the detected compounds is presented in Table 1, which includes the retention time, detected *m*/*z*, error value, molecular formula, and MS/MS data for identification purposes. From the ginseng tablet, a total of 39 chemical components were identified, while the ginseng residue contained 34 identified ginsenosides. Only ginsenoside Rg6, F4, Rs3, Rh2, and Compound K were detected in the ginseng tablet and were not found in the ginseng residue. The results indicated that after industrial extraction, most types of ginsenosides remain in ginseng residue. As displayed in Table 1, the contents of ginsenosides in the ginseng residue exhibited varying degrees of reduction, with ginsenoside content ranging from 0% to 54.38% of the corresponding compounds found in the ginseng tablet. Among the 39 types of ginsenosides, the residual amount of 30 ginsenosides in ginseng residue is below 30%. This result indicates that, to a certain extent, most of the ginsenosides have been extracted during the industrial extraction process. In order to further accurately determine the residual amount of total ginsenosides in ginseng residue, a UV-Vis method will be established to quantitatively measure the total ginsenosides in the ginseng residue and compare it with the total ginsenoside content in the ginseng tablet.

### 2.2. Quantitative Analysis of Total Ginsenosides and Total Polysaccharides in Ginseng Residue and Ginseng Tablet

**Figure 3:**
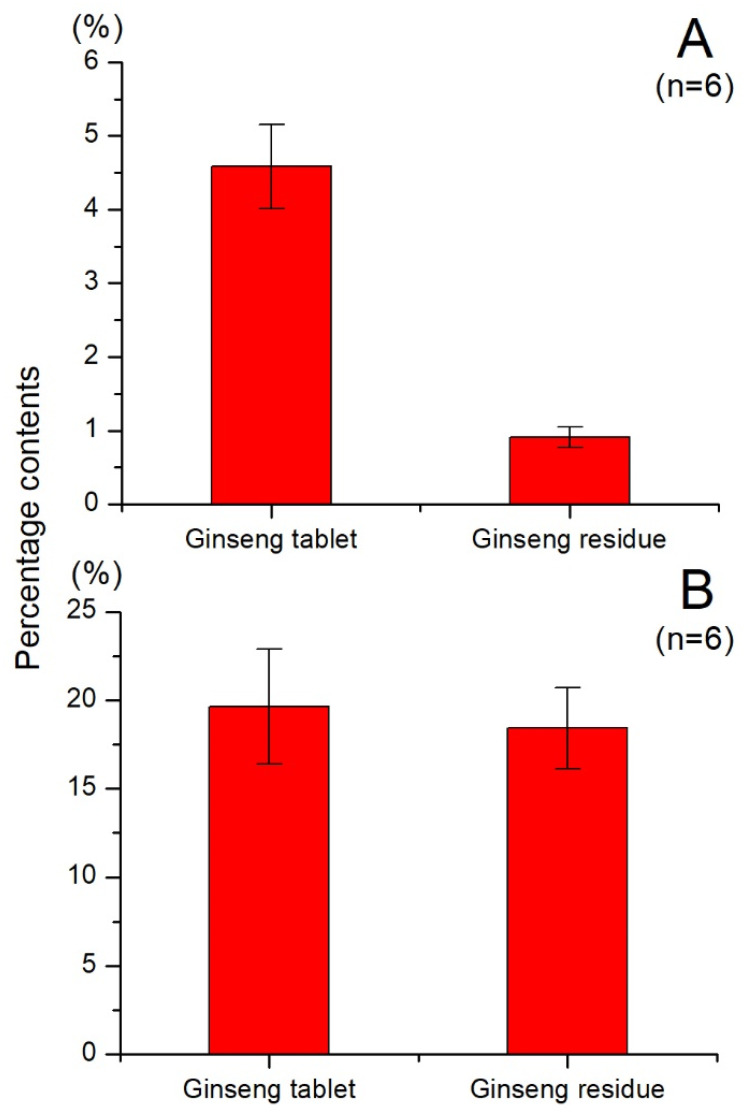
The contents of total ginsenosides (**A**) and total polysaccharides (**B**) in ginseng residue and ginseng tablet.

The analysis conducted using UPLC-Q-TOF MS revealed the presence of multiple ginsenosides in ginseng residue, which serve as important bioactive compounds in ginseng. To compare the variation in total ginsenoside content between ginseng residue and ginseng tablet, a UV-Vis method was employed for simultaneous determination. The equation of linear regression was y = 0.0049x + 0.1459 (*r* = 0.9964). The findings are depicted in Figure 3A. The content of total ginsenosides in the ginseng tablet was determined to be 4.58 ± 0.57%, whereas the content of total ginsenosides in the ginseng residue was found to be 0.91 ± 0.14%. These results indicate that only a small fraction of saponins remained in the ginseng residue, with more than 80% of the saponins being extracted. The result is consistent with the conclusion in Section 2.1, and they mutually validate each other, indicating that only a small amount of ginsenosides remains in the ginseng residue.

Polysaccharides represent another vital class of compounds contributing to the bioactivities of ginseng. To evaluate the disparity in total polysaccharide content between ginseng residue and ginseng tablet, a simultaneous determination was carried out using the UV-Vis method described in Section 3.3.2. The equation of linear regression was y = 12.589x + 0.1045 (*r* = 0.9957). The content of total polysaccharides in the ginseng tablets was determined to be 19.62 ± 3.25%, whereas the content of total polysaccharides in the ginseng residue was found to be 18.43 ± 2.29%. The total polysaccharides content in ginseng residue is approximately 94% of that in ginseng tablets. The results, as illustrated in Figure 3B, reveal that the content of total polysaccharides in ginseng residue is comparable to that of ginseng tablets. This suggests that the industrial production process extracts only a minimal amount of polysaccharides, resulting in their preservation within the ginseng residue.

The results of quantitative analysis of total ginsenosides and total polysaccharides in ginseng residue and ginseng tablets indicated that compared to ginseng tablets, ginseng residue contains a small number of ginsenosides and a large number of ginseng polysaccharides, which may still possess certain bioactivity that can be utilized. Therefore, the antioxidant activity was further investigated.

### 2.3. Evaluation of Antioxidant Activities of Ginseng Residue

The ABTS^+^ radical is widely employed for assessing the overall antioxidant activity in various samples. In the case of ginseng residue and ginseng tablet, their antioxidant effects demonstrated an increase in concentration ranging from 1.2 to 36 mg dried raw material equivalents/mL. To further evaluate these effects, IC50 values were utilized. It was observed that ginseng tablets exhibited greater antioxidant activity compared to ginseng residue (Table 2). The positive control drug Trolox and ginseng slices both exhibited excellent ABTS^+^ radical scavenging activity. The concentration of ginseng tablet extract required to achieve a 50% scavenging rate was 18.51 ± 2.38 mg/mL (mg dried raw material equivalents/mL), while the concentration of ginseng residue extract required to achieve a 50% scavenging rate was 23.46 ± 3.62 mg/mL. This indicated that the mass of ginseng residue required to achieve the same ABTS^+^ radical scavenging activity is 1.27 times that of ginseng tablets. Nevertheless, ginseng residue also demonstrated notable antioxidant activity. Considering the respective contents of ginsenosides and polysaccharides in ginseng residue, the antioxidant activity is primarily attributed to the presence of polysaccharides. According to previous reports [21,22], the ginseng polysaccharides, especially the ginseng neutral polysaccharide have good antioxidant activity, which supports the results of the evaluation of the antioxidant activities of ginseng residue. The results of the antioxidant activities of ginseng residue indicated the potential to develop ginseng residue into functional food or cosmetic products with antioxidant activity. The ginseng polysaccharides also exhibit anti-glioma [23], immunostimulatory [24], anticancer [25], antidiabetic [26], and phagocytic [27] activity. Considering the large amount of ginseng polysaccharide contained in ginseng residue, ginseng residue may also have the above activity, which needs further research and experimental support.

## 3. Materials and Methods

### 3.1. Chemical Reagents and Materials

HPLC-grade acetonitrile, methanol, and formic acid were procured from TEDIA (Fairfield, OH, USA), while ultrapure water was obtained using the Milli-Q water purification system (Millipore, Bedford, MA, USA). The ginsenoside Rb1, ginsenoside Re, ginsenoside Rg1, ginsenoside Rh1, ginsenoside Rh2, and ginsenoside Rg3 standards (abbreviated as Rb1, Re, Rg1, Rh1, Rh2, and Rg3, respectively, in the subsequent sections) were obtained from Chengdu Must Co. (purity: 99% HPLC, Chengdu, China). A total antioxidant capacity assay kit with the ABTS method was obtained from Shanghai Yuanye Bio-technology Co., Ltd. (Shanghai, China). Ginseng residue and ginseng tablets were provided by Changbaishan Pharmaceutical Co., Ltd. (Jilin, China). Ginseng residue refers to the residue left after the extraction of ginseng tablets with a 75% ethanol solution. The extraction process is as follows: add 20 times the amount of 75% ethanol solution to the ginseng tablets and simmer for 1 h. After filtration, the residue is added to an equal amount of 75% ethanol solution and simmered for 40 min. The residue is dried to obtain ginseng residue.

### 3.2. UPLC-Q-TOF/MS Analysis

To prepare a stock solution, standard references (Re, Rg1, Rb1, Rb2, Rb3, and Rg3) were accurately weighed and dissolved in 70% methanol. Method validation was facilitated by diluting the stock solutions of mixed standards to various concentrations using 70% methanol. After preparation, all solutions were stored at 4 °C. To prepare the samples, one gram of dried ginseng residue and black ginseng was ground into a powder using a mortar. Then, 10 mL of a 70% methanol aqueous solution was added. The mixture was subjected to ultrasonic extraction at room temperature for 60 min. After extraction, the resulting extract was filtered through a syringe filter with a pore size of 0.22 µm. The filtrate was subsequently injected directly into the UPLC-Q-TOF/MS system.

Chromatographic separation was performed using a Shimadzu LC 20 AD Prominence TM UPLC system, equipped with a Thermo Fisher Golden C18 column (2.1 × 50 mm, 1.9 µm), which was maintained at a temperature of 35 °C. Mobile phase A consisted of 0.1% formic acid in water, while mobile phase B consisted of 0.1% formic acid in ACN. The gradient for mobile phase B was programmed as follows: 15% (0 to 1 min), 15–40% (1 to 10 min), 40–95% (10 to 15 min), 95% (15 to 17 min), and 95–5% (17 to 17.1 min). The system was then equilibrated at 5% for 2.9 min. The injection volume was set to 5.0 µL, and a flow rate of 0.3 mL/min was employed.

For Q-TOF/MS detection, a Triple-TOF 5600+ MS instrument (SCIEX, Concord, ON, Canada) with an electrospray ionization source was utilized. The MS parameters were configured for negative ion mode, with an electrospray ionization source temperature of 500 °C, an ion spray voltage of −4500 V, nebulizer gas (N_2_) at 55 psi, heater gas (N_2_) at 60 psi, and curtain gas (N_2_) at 35 psi. The declustering potential was set to 100 V. Full-scan MS data were acquired in TOF/MS mode, covering a mass range of *m*/*z* from 100 to 1500 with a collision energy of 5 eV. For MS/MS acquisition, the IDA mode was used with a collision energy of 35 eV and a rolling collision energy of 15 eV. The mass range for both MS and MS/MS spectra was from *m*/*z* 100 to 1500.

### 3.3. Quantitative Analysis

#### 3.3.1. Quantitative Analysis of Total Ginsenosides

The ginseng residue and ginseng tablet samples were ground into a powder. A 1.0 g portion of the sample powder was accurately weighed and wrapped in neutral filter paper. The paper-wrapped sample was placed in a reflux extraction device, and ether was added. The extraction was carried out at approximately 38 °C with micro-boiling reflux for 1 h. The ether was then discarded, and the sample was dried in a medicine bag before being soaked in methanol overnight. The following day, the methanol in which the sample was soaked was directly placed in a Soxhlet extractor. An additional amount of methanol was added, and reflux extraction was conducted at 80 °C for 4 h, with approximately one siphon extraction per hour. The methanol extracts were combined and concentrated to a trace amount using a rotary evaporator. The concentrated extract was transferred to an evaporation dish, and the container was washed with a small amount of methanol, which was then transferred to the evaporation dish. The methanol was dried at 60 °C. After drying, the extract was dissolved in 30 mL of distilled water in a separating funnel. The mixture was extracted four times with 50 mL of n-butanol. The combined extraction liquid was concentrated to a trace amount using a rotary evaporator and then dried. The resulting product was dissolved in methanol, washed, and transferred to a 10 mL volumetric bottle. The volume was adjusted to a constant volume with methanol, resulting in the test solution. A precise amount of ginsenoside Re was accurately weighed and added to the standard material solution, which was prepared by dissolving 2 mg/mL in methanol. The solution was then diluted to various concentrations to obtain the standard working solution.

A 10 μL volume of the standard working solution was placed into a glass tube with a stopper. Dry methanol was added to the tube, followed by 5 mL of 72% concentrated sulfuric acid. Then, 0.5 mL of an 8% anhydrous ethanol solution of vanillin was added. The mixture was shaken well and placed in a constant temperature water bath at 60 °C for 10 min. Afterward, it was immediately cooled with ice water for 10 min and shaken well. The absorbance was then measured at 544 nm using a mixture of concentrated sulfuric acid and vanillin anhydrous ethanol as a blank control. A standard curve was constructed using the absorbance as the *y*-axis and the ginsenoside concentration as the *x*-axis. The same procedure was performed on the sample working solution, and the obtained absorbance value was substituted into the standard curve to calculate the total ginsenoside content.

#### 3.3.2. Quantitative Analysis of Total Polysaccharides

One gram of the sample powder was accurately weighed and then soaked in 50 times its volume of water overnight. It was decocted three times for 2 h each time. The filtrate obtained after filtration was combined from the three decoctions. The combined filtrate was then concentrated under reduced pressure. Next, 90% ethanol was added, and it was left to stand overnight. The precipitate was collected, and 90% ethanol was added to the supernatant. After an hour of standing, it was centrifuged at 13,000 rpm for 10 min. The resulting precipitate was dried at 60 °C to obtain the crude extract of ginseng polysaccharide.

A certain amount of the ginseng polysaccharide crude extract was dissolved in water to obtain a 50 μg/mL solution. Then, 2 mL of the above solution was taken and 1 mL of 5% phenol was added. This was followed by the addition of 5 mL of concentrated sulfuric acid. The mixture was shaken well and left at room temperature for 10 min. It was then heated in a water bath at 40 °C for 15 min, cooled to room temperature, and the absorbance value was measured at a wavelength of 490 nm. Similarly, a series of glucose standard solutions with different concentrations were treated using the same method, and their absorbance values were determined.

### 3.4. ABTS^+^ Radical Scavenging Activity

Ginseng residue and ginseng tablet samples were boiled three times in twenty times their volume of water, with each boil lasting 40 min. The resulting extraction solutions were combined. These solutions were then concentrated and freeze dried to obtain extracts from both the ginseng residue and ginseng tablets. A certain quantity of the extract was dissolved in water and further diluted to create sample solutions with different concentrations. These solutions were used to determine the antioxidant activity.

To begin, a solution of reagent A (ABTS^+^ reagent) from the total antioxidant capacity assay kit was mixed with reagent B (oxidizing agent) and kept in the dark at room temperature for 14 h. The solution was then diluted with water to obtain a working solution. Next, the sample solution (7 μL) was mixed with 280 μL of the ABTS^+^ working solution and the mixed solution was kept in the dark at room temperature for 6 min. Then, the absorbance of the post-reaction solution was detected at 734 nm using a microplate reader. For the blank group, 280 μL of water was mixed with 7 μL of the sample solution, replacing the ABTS^+^ solution. The control group was created by mixing 7 μL of water with 280 μL of the ABTS^+^ solution. Trolox is an analog of vitamin E, possessing a similar antioxidant capacity to vitamin E, and can be used as a reference for the total antioxidant capacity of other antioxidants. Trolox was used as a positive control. The scavenging activity of the ABTS^+^ radical cation was calculated using the following equation:

$$ABTS\cdot^{+}radical\;scavenging\;activity\left(\%\right)=\left[1-\frac{A_{s}-A_{0}}{A_{1}}\right]\times 100\%$$

In the equation provided, A*_S_* represents the absorbance of the sample group, A_0_ represents the absorbance of the blank group, and A_1_ represents the absorbance of the control group.

The IC50 values for both the ABTS^+^ radical scavenging activity were determined using SPSS 19.0. All experiments were performed in triplicate, and the IC50 values were determined through a probit regression analysis.

## 4. Conclusions

In this study, a comprehensive analysis utilizing the UPLC-Q-TOF/MS method revealed a total of 39 ginsenosides in both ginseng residue and ginseng tablets. Notably, only ginsenosides Rg6, F4, Rs3, Rh2, and Compound K were detected in the ginseng tablets. These chemical profiles obtained from our research play a vital role in distinguishing ginseng residue from ginseng tablets. Through quantitative analysis, it was observed that the industrial production process primarily extracts most of the ginsenosides while leaving behind only a small quantity of polysaccharides. The residual polysaccharides in ginseng residues provide a material basis for its excellent bioactivity. Remarkably, despite this discrepancy, the ginseng residue displayed a similar antioxidant effect to the ginseng tablet. These findings establish a reliable theoretical foundation and scientific basis for the potential reuse of ginseng residue. The market price of ginseng residue is less than 5% of ginseng tablets. Therefore, compared to ginseng tablets, developing products related to ginseng residue has enormous economic value. This study also provides new research ideas and methods for the protection and recycling of traditional Chinese medicine resources, as well as the development of related products.

## Figures and Tables

**Table 1:** Qualitative analysis of ginseng residue and ginseng tablet using UPLC-Q-TOF MS.

No.	RT (min)	Measured *m*/*z*	Mass Deviation (ppm)	Adducts	Product Ions	Compounds	Formula	Peak Area of Ginseng Residue/Ginseng Tablet (%)
1	3.79	1007.5534	10.12	[M+HCOO]^−^	961.5574, 799.4996, 637.4438	20-Glc-Rf	C48H82O19	19.82
2	4.52	977.5421	9.62	[M+HCOO]^−^	931.5450, 799.4996, 637.4402	Noto R1	C47H80O18	24.54
3 *	6.37	845.4956	6.15	[M+HCOO]^−^	799.5001, 637.4424, 475.3846	Rg1	C42H72O14	17.78
4 *	6.86	991.5559	7.66	[M+HCOO]^−^	945.5588, 799.4996, 783.5050, 637.4418	Re	C48H82O18	18.59
5	8.43	827.4838	4.83	[M+HCOO]^−^	781.4884, 619.4333	Rg9/Rg8	C42H70O13	3.60
6	9.82	1031.5517	8.24	[M-H]^−^	987.5748, 945.5434, 927.5525, 799.5008	mRe	C51H84O21	5.44
7	12.48	845.496	6.62	[M+HCOO]^−^	799.4997, 637.4435, 475.3860	Rf	C42H72O14	17.50
8	13.08	913.5222	6.13	[M+HCOO]^−^	867.5276, 799.5000, 781.4883	Re6	C46H76O15	46.35
9	13.27	815.4847	6.01	[M+HCOO]^−^	769.4891, 637.4419, 475.3846	Noto R2	C41H70O13	23.06
10	13.95	1285.656	9.8	[M+HCOO]^−^	1239.6656, 1077.6281	Ra3	C59H100O27	37.64
11	14.07	683.4413	5.41	[M+HCOO]^−^	637.4407, 475.3801	F1	C36H62O9	17.09
12	14.24	829.4988	3.98	[M+HCOO]^−^	783.5053, 637.4469, 475.3846	Rg2	C42H72O13	13.50
13	14.33	1325.6462	4.67	[M-H]^−^	1325.6462	mRa3	C62H102O30	10.05
14^#^	14.59	811.491	7.52	[M+HCOO]^−^	769.4871, 619.4269, 475.3824	Rg6	C42H70O12	0
15	15.08	1255.6408	6.37	[M+HCOO]^−^	1209.5873, 1077.6061	Ra1	C58H98O26	32.97
16 *	15.27	1153.6123	9.71	[M+HCOO]^−^	1107.6182, 945.5630, 783.5052	Rb1	C54H92O23	26.63
17	15.65	1193.6003	3.52	[M-H]^−^	1149.6313, 1107.6206, 1089.6096, 945.5598, 783.5021	mRb1	C57H94O26	10.00
18	15.83	1123.6002	8.54	[M+HCOO]^−^	1077.6097, 945.5621	Rc	C53H90O22	27.39
19	15.96	1255.6428	7.96	[M+HCOO]^−^	1209.6536, 1077.6084	Ra2	C58H98O26	54.38
20	16.07	1001.5011	4.79	[M+HCOO]^−^	955.5147, 793.4542	Ro	C48H76O19	49.06
21	16.21	1163.5952	8.34	[M-H]^−^	1119.6217, 1077.6107, 1059.5996	mRc	C56H92O25	6.49
22	16.22	1165.6098	7.46	[M+HCOO]^−^	1119.6184, 1077.6069, 1059.5966	Rs1	C55H92O23	3.74
23	16.29	1295.6326	3.7	[M-H]^−^	-	m Ra1/m Ra2	C61H100O29	7.26
24 *	16.45	1123.6003	8.63	[M+HCOO]^−^	1077.6072, 945.5597, 915.5481, 783.5034	Rb2	C53H90O22	46.33
25 *	16.69	1123.6009	9.17	[M+HCOO]^−^	1077.6079, 945.5579, 915.5535, 783.5058	Rb3	C53H90O22	15.42
26	16.82	1163.5913	4.98	[M-H]^−^	1119.6184, 1077.6080, 1059.5979	mRb2	C56H92O25	6.45
27	17.07	1163.5911	4.81	[M-H]^−^	1119.6178, 1077.6052, 1059.5935	mRb3	C56H92O25	5.03
28	17.66	991.5568	8.57	[M+HCOO]^−^	945.5615, 783.5049, 621.4464	Rd	C48H82O18	25.23
29	18.04	1031.5512	7.76	[M-H]^−^	987.5718, 945.5615, 927.5497	mRd	C51H84O21	12.77
30 ^#^	20.56	811.4897	5.92	[M+HCOO]^−^	765.4939, 619.4329	F4	C42H70O12	0
31	20.91	665.4322	7.81	[M+HCOO]^−^	619.3430, 341.1122, 295.2295	Rh4	C36H60O8	47.37
32	21.1	811.4905	6.9	[M+HCOO]^−^	765.4932, 619.4310, 570.7531	Rk1	C42H70O12	7.91
33	21.85	829.5016	7.35	[M+HCOO]^−^	783.5039, 621.4471	F2	C42H72O13	3.79
34	23.03	839.4473	4.53	[M+HCOO]^−^	793.4514	zingibroside	C42H66O14	40.93
35 *	23.37	829.5008	6.39	[M+HCOO]^−^	783.5044, 621.4603	Rg3	C42H72O13	26.88
36 ^#^	23.45	871.5122	6.99	[M-H]^−^	825.5154, 783.5054	Rs3	C44H74O14	0
37 ^#^	24.06	667.4484	8.54	[M+HCOO]^−^	621.4453	Rh2	C36H62O8	0
38	24.11	811.4878	3.57	[M+HCOO]^−^	765.4916, 603.4352	Rg5	C42H70O12	38.56
39 ^#^	24.83	667.4473	6.89	[M+HCOO]^−^	333.2319, 275.1532	Compound K	C36H62O8	0

#: The compound was found only in ginseng tablets in ginseng residue. *: The identification was confirmed by standard.

**Table 2:** IC50 values of Du-Zhong tea extracts for ABTS^+^ radical scavenging.

Sample	Trolox (mM)	Ginseng Tablet Extracts (mg Dried Raw Material Equivalents/mL)	Ginseng Residue Extracts (mg Dried Raw Material Equivalents/mL)
IC50 values	0.91 ± 0.04	18.51 ± 2.38	23.46 ± 3.62

## Data Availability

The data presented in this study are available upon request from the corresponding author.

## References

[1] Liu C.-X., Xiao P.G. (1992). Recent advances on ginseng research in China. J. Ethnopharmacol..

[2] Wu W., Song F., Guo D., Mi J., Qin Q., Yu Q., Liu S. (2012). Mass Spectrometry-Based Approach in Ginseng Research: A Promising Way to Metabolomics. Curr. Anal. Chem..

[3] Jin Y., Kim Y.-J., Jeon J.-N., Wang C., Min J.-W., Noh H.-Y., Yang D.-C. (2015). Effect of White, Red and Black Ginseng on Physicochemical Properties and Ginsenosides. Plant Foods Hum. Nutr..

[4] Lee M.-R., Yun B.-S., Sung C.-K. (2012). Comparative Study of White and Steamed Black *Panax ginseng*, *P. quinquefolium*, and *P. notoginseng* on Cholinesterase Inhibitory and Antioxidative Activity. J. Ginseng Res..

[5] Lee W.-J., Shin Y.-W., Chang H., Shin H.-R., Kim W.-W., Jung S.-W., Kim M., Nah S.-Y. (2021). Safety and efficacy of dietary supplement (gintonin-enriched fraction from ginseng) in subjective memory impairment: A randomized placebo-controlled trial. Integr. Med. Res..

[6] Kocak M.Z., Aktas G., Bilgin S., Duman T.T., Kurtkulagi O., Atak B., Demirkol M.E., Yilmaz E.B. (2020). An unusual immune thrombocytopenia case associated with dietary supplements containing 3G (Green tea, Ginseng and Guarana). Russ. Open Med. J..

[7] Riaz M., Rahman N.U., Zia-Ul-Haq M., Jaffar H.Z., Manea R. (2018). Ginseng: A dietary supplement as immune-modulator in various diseases. Trends Food Sci. Technol..

[8] Truong V.-L., Jeong W.-S. (2021). Red ginseng (*Panax ginseng* Meyer) oil: A comprehensive review of extraction technologies, chemical composition, health benefits, molecular mechanisms, and safety. J. Ginseng Res..

[9] Zhang K., Zhang S., Ebihara A., Zhou X., Fan L., Li P., Zhang Z., Wang Y., Shen Y. (2023). The current research progress of ginseng species: The cultivation and application. Cogent Food Agric..

[10] Tao R., Lu K., Zong G., Xia Y., Han H., Zhao Y., Wei Z., Lu Y. (2023). Ginseng polysaccharides: Potential antitumor agents. J. Ginseng Res..

[11] Qi X., Lu X., Han Y., Xing Y., Zheng Y., Cui C. (2023). Ginseng polysaccharide reduces autoimmune hepatitis inflammatory response by inhibiting PI3K/AKT and TLRs/NF-κB signaling pathways. Phytomedicine.

[12] Zhang R., Lv C., Lu J. (2020). Studies on laccase mediated conversion of lignin from ginseng residues for the production of sugars. Bioresour. Technol..

[13] Sun J., Zhong X., Sun D., Cao X., Yao F., Shi L., Liu Y. (2022). Structural characterization of polysaccharides recovered from extraction residue of ginseng root saponins and its fruit nutrition preservation performance. Front. Nutr..

[14] Shi J., Gao X., Zhang A., Qin X., Du G. (2021). Characterization of multiple chemical components of GuiLingJi by UHPLC-MS and 1H NMR analysis. J. Pharm. Anal..

[15] Guo P., Zhong F., Zhao Y., Xu X., Xue W., Wang Y., Song X., Tang W., Fan D. (2022). Thermosensitive molecularly imprinted polymer coupled with HPLC for selective enrichment and determination of matrine in traditional Chinese medicine. J. Chromatogr. B.

[16] Li L., Ma L., Guo Y., Liu W., Wang Y., Liu S. (2019). Analysis of oligosaccharides from *Panax ginseng* by using solid-phase permethylation method combined with ultra-high-performance liquid chromatography-Q-Orbitrap/mass spectrometry. J. Ginseng Res..

[17] Li Z.-T., Zhang F.-X., Fan C.-L., Ye M.-N., Chen W.-W., Yao Z.-H., Yao X.-S., Dai Y. (2021). Discovery of potential Q-marker of traditional Chinese medicine based on plant metabolomics and network pharmacology: Periplocae Cortex as an example. Phytomedicine.

[18] Shi X., Luo S., Zhong K., Hu X., Zhang Z. (2022). Chemical profiling, quantitation, and bioactivities of Du-Zhong tea. Food Chem..

[19] Qiu S., Yang W.-Z., Shi X.-J., Yao C.-L., Yang M., Liu X., Jiang B.-H., Wu W.-Y., Guo D.-A. (2015). A green protocol for efficient discovery of novel natural compounds: Characterization of new ginsenosides from the stems and leaves of *Panax ginseng* as a case study. Anal. Chim. Acta.

[20] Wu W., Sun L., Zhang Z., Guo Y., Liu S. (2015). Profiling and multivariate statistical analysis of *Panax ginseng* based on ultra-high-performance liquid chromatography coupled with quadrupole-time-of-flight mass spectrometry. J. Pharm. Biomed. Anal..

[21] Chen F., Huang G. (2018). Antioxidant activity of polysaccharides from different sources of ginseng. Int. J. Biol. Macromol..

[22] Kim H.M., Song Y., Hyun G.H., Long N.P., Park J.H., Hsieh Y.S., Kwon S.W. (2020). Characterization and Antioxidant Activity Determination of Neutral and Acidic Polysaccharides from *Panax Ginseng* C. A. Meyer. Molecules.

[23] Cao D., Wei E., Wang Z., Hu Z., Qi L., Zhou H., Zhao J. (2022). Microwave-assisted extraction, structural elucidation, and in vitro anti-glioma and immunostimulatory activity of polysaccharide from *Panax ginseng* C. A. Meyer. Ind. Crop. Prod..

[24] Akhter K.F., Mumin A., Lui E.M., Charpentier P.A. (2018). Fabrication of fluorescent labeled ginseng polysaccharide nanoparticles for bioimaging and their immunomodulatory activity on macrophage cell lines. Int. J. Biol. Macromol..

[25] Zhai F.-G., Liang Q.-C., Wu Y.-Y., Liu J.-W. (2022). Red ginseng polysaccharide exhibits anticancer activity through GPX4 downregulation-induced ferroptosis. Pharm. Biol..

[26] Li J., Li R., Li N., Zheng F., Dai Y., Ge Y., Yue H., Yu S. (2018). Mechanism of antidiabetic and synergistic effects of ginseng polysaccharide and ginsenoside Rb1 on diabetic rat model. J. Pharm. Biomed. Anal..

[27] Reyes A.W.B., Simborio H.L.T., Hop H.T., Arayan L.T., Min W.G., Lee H.J., Rhee M.H., Chang H.H., Kim S. (2016). Inhibitory effect of red ginseng acidic polysaccharide from Korean red ginseng on phagocytic activity and intracellular replication of *Brucella abortus* in RAW 264.7 cells. J. Vet.-Sci..

